# Neurons in the Locus Coeruleus Modulate the Hedonic Effects of Sub-Anesthetic Dose of Propofol

**DOI:** 10.3389/fnins.2021.636901

**Published:** 2021-03-09

**Authors:** Hui Chen, Dan Xu, Yu Zhang, Yan Yan, JunXiao Liu, ChengXi Liu, Wei Shen, Tian Yu, Jin Liu

**Affiliations:** ^1^Department of Anesthesiology, West China Hospital, Sichuan University, Chengdu, China; ^2^Guizhou Key Laboratory of Anesthesia and Organ Protection, Zunyi Medical University, Guizhou, China; ^3^School of Life Sciences and Technology, ShanghaiTech University, Shanghai, China

**Keywords:** propofol, hedonic, locus ceruleus, ventral tegmental area, chemogenetic, fiber photometry

## Abstract

Propofol is a worldwide-used intravenous general anesthetic with ideal effects, but hedonic effects of propofol have been reported and cause addictive issue. There is little known about the neurobiological mechanism of hedonic effects of propofol. Increasing researches have shown that the dopaminergic nervous system of the ventral tegmental area (VTA) and the noradrenergic system of locus coeruleus (LC) play a crucial role in hedonic experiences, which are putative sites for mediating the hedonic effects of propofol. In the present study, rat hedonic response scale and place conditioning paradigm were employed to examine the euphoric effects of propofol. *In vivo* GCaMP-based (AVV-hSyn-GCaMP6s) fiber photometry calcium imaging was used to monitor the real-time neuronal activity in VTA and LC area in rats exhibiting propofol-induced euphoric behaviors. Then DREADDs (designer receptors exclusively activated by designer drugs) modulation using rAAV-hSyn-hM4D(Gi)-EGFP was performed to confirm the neuronal substrate that mediates the euphoric effects of propofol. The score of hedonic facial responses was significantly increased in the 4 mg/kg group compared with that of the 0 mg/kg group. The locomotor activity in the propofol-paired compartment was significantly increased at the 4 mg/kg dose compared with that of the saline-paired group. When compared with the 0 mg/kg group, the place preference increased in the 4 mg/kg group. Administration of 4 mg/kg of propofol triggers reliable increases in GcaMP fluorescence. However, in the VTA GcaMP-expressing rats, administration of 4 mg/kg of propofol did not induce any change of GcaMP signals. The facial score and the place preference, which increased by 4 mg/kg propofol were abolished by chemogenetic inhibition of the neuronal activity in the LC area. Our results suggest that LC noradrenergic neurons, not VTA dopaminergic neurons, are directly involved in the hedonic effects of sub-anesthetic dose of propofol.

## Introduction

Propofol is the most commonly used intravenous general anesthetic that acts by facilitating the inhibitory neurotransmission mediated by gamma-aminobutyric acid (GABA). Propofol can directly activate the GABA_*A*_ receptor or, when coapplied with GABA or other agonists, potentiate the response of GABA_*A*_ receptor to the transmitter (Hales and Lambert, 1991; Shin et al., 2018). In addition to being commonly used for the maintenance of anesthesia in patients undergoing long-term surgery, propofol is also used for short procedures such as painless gastrointestinal endoscopy due to its short-acting characteristics. Propofol has no obvious respiratory and circulatory side effects, but various hedonic effects such as feelings of wellbeing, sexual hallucinations, and euphoria have been reported (Tezcan et al., 2014). Clinical study observed and evaluated the postoperative experience of 82 patients undergoing gastroenteroscopy under propofol anesthesia and found that 43.9% of the patients experience obvious euphoria during the period of anesthesia recovery (Brechmann et al., 2018).

The euphoric and addictive effects of the low-dose propofol can be reflected by animal conditional location preference test or self-administered test (Pain et al., 1996), but its specific neurobiological mechanism is still not fully understood. Some studies have focused on the dopaminergic nervous system of the ventral tegmental area (VTA), which is also known as the central reward system (Berridge and Kringelbach, 2015). It has been found that a very low concentration of propofol can activate glutamate transmission to dopamine neurons in the VTA (Li et al., 2008). The dopamine D1 receptors in the nucleus accumbens were found to mediate the propofol-induced self-administration in rats (Lian et al., 2013). However, there is no direct evidence proving that propofol can affect the activity of dopaminergic neurons in VTA. Furthermore, an *in vivo* microdialysis experiment showed that propofol decreased the dopamine level in the ventral pallidum of free active rats (Grasshoff et al., 2005). Our previous study also found that propofol significantly reduced the level of dopamine in the prefrontal cortex of rats (Wang et al., 2016b). These results do not support the idea that the euphoric effects of propofol are mediated by the dopaminergic nervous system.

In addition to the dopaminergic system, the locus coeruleus (LC) noradrenergic system also plays an important role in the regulation of reward behavior. LC is considered to function as a relay station and has widespread projections throughout the brain (Devilbiss et al., 2006). Many regions of the mesolimbic reward pathways receive noradrenergic input (Guiard et al.,2008a,b; Gonzalez et al., 2012). It was reported that the activation of noradrenergic neurons in the LC enhances the activity of dopaminergic neurons in the VTA (Grenhoff et al., 1993). Norepinephrine was found necessary for morphine-induced conditioned place preference and locomotion (Olson et al., 2006), and noradrenergic neurons of the LC were found to be important components of the nicotine reward circuitry (Rose et al., 2016). Thus, the LC is a putative site for mediating the euphoric effects of propofol.

The current study was therefore designed to study the role of the VTA neurons and LC neurons in the euphoric state induced by propofol. For this purpose, rat hedonic response scale and place conditioning paradigm were employed to examine the euphoric effects of propofol. *In vivo* GCaMP6-based fiber photometry calcium imaging was used to measure real-time neuronal activity in the VTA and LC in rats exhibiting propofol-induced euphoric behaviors. In the final step, we used DREADDs (designer receptors exclusively activated by designer drugs) technology to define the neural function of the VTA or LC to the euphoric effects of propofol. We report that propofol-induced euphoric behaviors are not associated with the activity of VTA neurons, whereas LC neurons appear to play an more important role in regulating the euphoric effects of propofol.

## Materials and Methods

### Animals

Male 12- to 16-week-old Sprague–Dawley rats, initially obtained from the animal center of the third military medical university (Chongqing, China), were bred in the Guizhou Key Laboratory of Anesthesia and Organ Protection in Zunyi Medical University. All rats were maintained on a 12/12-h light/dark cycle at 21–25°C with free access to water and food. All behavioral manipulations were performed during the light cycle. All experiments were conducted in accordance with the guidelines of the Animal Care and Use Committees of Zunyi Medical University.

### Hedonic Responses Scoring

A preliminary study has been conducted to determine the hedonic facial reactions made by rats (Okun et al., 2016). In general, rats were placed in an acrylic glass chamber (5 × 6 × 6 inches) with a tail hole for tail-vein injection. Propofol (Fresenius Kabi, Beijing, China) was delivered via tail-vein bolus injection (i.v.) at five different doses: 0, 2, 4, 6, and 8 mg/kg. According to the dose of propofol, 60 rats were randomly assigned to five groups (*n* = 12). The group of 0 mg/kg received a volume of vehicle equivalent to the volume of propofol of the other groups. After the propofol bolus injection, a 5-min period of facial reactions was recorded by a digital camera (Cannon, Japan) positioned underneath the chamber and pointed at the ventral side of the rats. The facial and body responses of the rats in the videos were analyzed and scored by a trained observer who was blinded to the propofol usage. For accurate scoring, the speed of the video was reduced to 1/10th of the actual speed. Paw licks, rhythmic midline tongue protrusions, and lateral tongue protrusions were defined as hedonic responses and scored with 1 point each time that they occurred. If paw licks and rhythmic midline tongue protrusions occur for >5 s, it scored with 2 points.

### Conditioned Place Preference (CPP)

Three weeks after the hedonic response test, the rats in five groups carried out the CPP procedure. The place-conditioning apparatus consisted of three Plexiglas boxes: two equal-sized compartments (30 cm long × 30 cm wide × 45 cm high) separated by a gray partition. One compartment had a black wall, and the other one had a white wall. An Infrared digital camera fixed 70 cm above the apparatus was used to record the position of the rats. The camera was connected to a Lenovo PC computer using Smart 3.0 software (Panlab Harvard Apparatus, Shanghai, China).

The CPP consisted of three consecutive phases and conducted between 12:00 and 6:00 PM. The first phase is pre-conditioning. Each rat was placed into the gray partition and allowed to move freely to the two compartments. The time spent by the rat in each compartment during a 15-minute period was recorded. Rats showing a preference for one compartment or obvious unconditioned aversion were excluded from the study. The second phase is conditioning, which lasted for 4 days. Each rat in the five groups was injected with saline before being confined to the vehicle-paired compartment for 30 min and, after an interval of 4 h, received propofol before being confined to the drug-paired compartment for 30 min. The third phase is post-conditioning. Each rat in five groups with no treatment was placed into the gray partition and allowed to move freely to the two compartments. The time spent by the untreated rats in each compartment during a 15-minute period was recorded.

### Fiber Photometry

Fiber photometry was conducted as previously described (Luo et al., 2018). AVV-hSyn-GCaMP6s (500 nl) (BrainVTA Co., Ltd., WuHan, China) was injected into the right VTA (AP: −4.80 mm, ML:0.9 mm, DV: −8.3 mm) or the right LC (AP: −9.84 mm, ML: −1.4 mm, DV: −7.0 mm) and allowed 4 weeks for sufficient virus expression. Optic fibers (OD: 200 μm, numerical aperture: 0.37; Newton Inc., Hangzhou, China) were implanted targeting the right VTA or LC to transmit signals for fiber photometry. Optic fiber was connected to a multi-channel fiber photometry system (ThinkerTech Nanjing Bioscience Nanjing, China) equipped with a LED light (*λ* = 480 nm) and a dichroic mirror (DCC3420M; Thorlabs). LED was controlled by an LED driver and computer running a multifunction data acquisition software (Thinker Tech Nanjing Bioscience Inc.). Rats expressing GCaMP in VTA (*n* = 8) or LC (*n* = 8) were habituated in a glass chamber, which is the same as for the hedonic response test for 15 min before recording. The recording protocol lasted for 100 s and consisted of 50-s pre-propofol and 50-s post-propofol periods. During each period, GCaMP signals of spontaneous activity were recorded. Propofol was delivered via tail-vein bolus injection (i.v.) at five different doses: 0, 2, 4, 6, and 8 mg/kg. Brain tissue was harvested after the recordings for validation of virus expression and optic fiber locations. Data were analyzed using MATLAB (R2016a). The normalized values of GCaMP signals (ΔF/F, expressed as percentages) were calculated using the following formula: (F−F0)/F0, where F0 is the baseline GCaMP signal averaged over a window of 20 s, and F is the real-time GCaMP signal.

### Chemogenetic Inhibition of Ventral Tegmental Area or Locus Coeruleus Neuron and Behavioral Test

Chemogenetic inhibition generally followed our previous study (Zhang et al., 2019). AAV vectors containing the Gi-coupled inhibitory human M4 muscarinic receptor (rAAV-hSyn-hM4D(Gi)-EGFP, BrainVTA Co., Ltd., WuHan, China) were injected in the target area and allowed 4 weeks for sufficient virus expression. Then we conducted hedonic responses scoring and CPP test in two groups of rats. In group 1, six rats expressing hM4D(Gi) in the target area were intraperitoneally injected with clozapine N-oxide (CNO, 1 mg/kg), which allowed specific inhibition of hM4D(Gi) expressing neurons. Thirty minutes after the injection of CNO, hedonic responses scoring of propofol administration were conducted as mentioned above. In group 2, new six rats expressing hM4D(Gi) in the target area were intraperitoneally injected with CNO. After 30 min, the CPP test following a single-dose propofol administration was conducted as mentioned above. The dose of propofol that induced the most obvious positive reaction in previous hedonic response scoring and CPP test was chosen for the chemogenetic inhibition experiments. Brain tissue was harvested after the tests for validation of virus expression.

### Histology

Rats were transcardially infused with 300 ml of 0.01 M PBS, followed by 250 ml of 4% PFA. The brain was removed and fixed in 4% PFA overnight at 4°C, later transferred to 30% sucrose at 4°C until they sank. A freezing microtome (CM1950; Leica, Germany) was used to collect 30-μm-thick coronary brain slices. Then the slices were imaged with a 4× and 10× objective on a BX63 fluorescence microscope (Olympus, Japan) to validate the virus sites.

### Statistical Analysis

All statistical analyses were performed by the GraphPad Prism software package, version 6.0 (GraphPad Software Inc., CA, United States). All data were subject to tests for normality. One-way ANOVAs were used to compare the differences in hedonic response scoring and CPP time between the different groups. Furthermore, the paired Student’s *t*-tests were used to analyze the differences in calcium signals between the pre- and post-events. Data are presented as mean ± SEM. In all cases, *P*-values <0.05 were considered significant.

## Results

### Sub-Anesthetic Dose of Propofol Induce Facial Hedonic Responses

**FIGURE 1:**
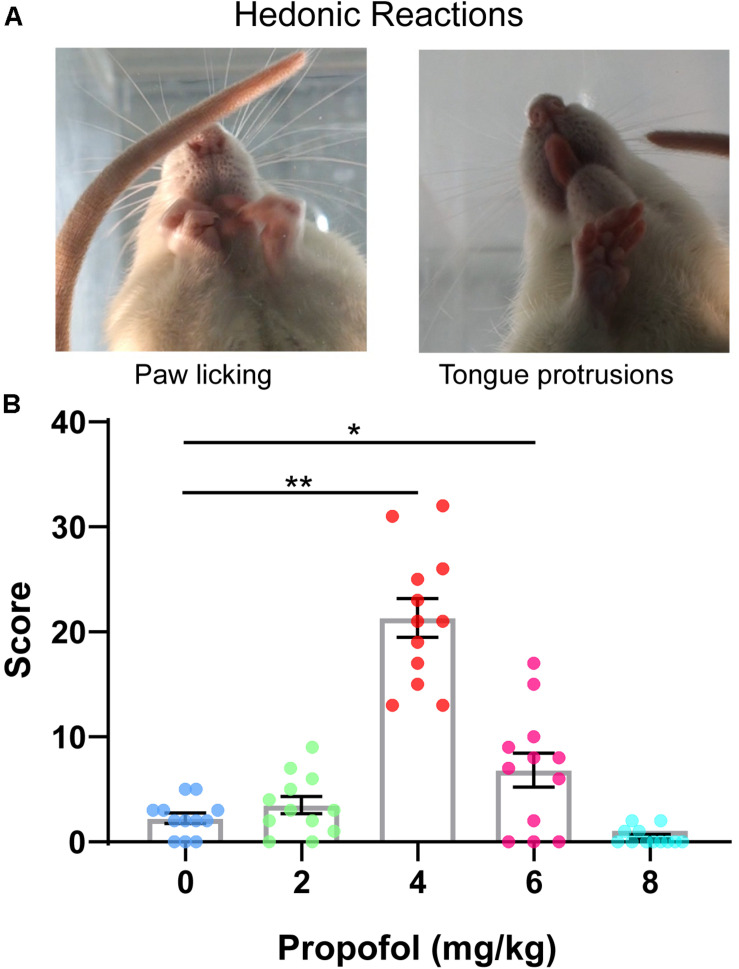
Scoring of hedonic facial responses. **(A)** Representative examples of hedonic facial responses. **(B)** Hedonic facial responses were scored according to the facial reactivity scale. 4 mg/kg group showed the greatest level of hedonic reactions to propofol compared with 0 mg/kg group (*P < 0.05, **P < 0.01, One-way ANOVAs). No difference in hedonic reactivity was detected between 0, 2, and 8 mg/kg groups. All data are graphed as mean ± SEM.

To investigate whether the administration of propofol induces hedonic responses, facial responses during 0–5 min after 0, 2, 4, 6, and 8 mg/kg of propofol tail-vein bolus injection were assessed. Hedonic facial responses like paw licks and tongue protrusions are shown in Figure 1A. The score of hedonic facial responses was not significantly different between the 0 and 2 mg/kg groups (2.25 ± 0.49 vs. 3.50 ± 0.81) (Figure 1B). As expected, the score of hedonic facial responses was significantly increased in the 4 mg/kg groups (21.33 ± 1.84 vs. 2.25 ± 0.49, *P* < 0.01) (Figure 1B). Additionally, 8/12 rats in the 8 mg/kg group were anesthetized and showed no autonomous activities. The other four rats showed no increase in the score of hedonic facial responses. Collectively, hedonic facial responses can be induced by sub-anesthetic dose of propofol, not anesthetic dose of propofol.

### Sub-Anesthetic Dose of Propofol Induced Conditioned Place Preference

**FIGURE 2:**
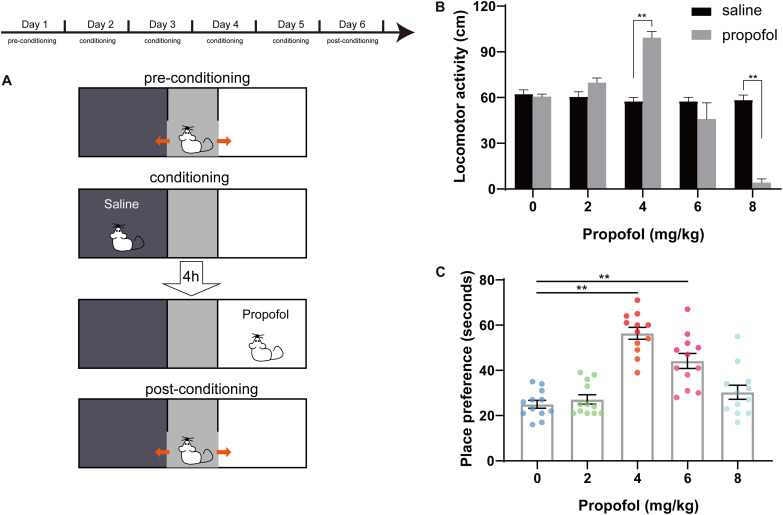
Conditioned place preference (CPP) design and effects of propofol at five doses on CPP. **(A)** Pre-conditioning phase. Conditioning sessions and post-conditioning phase. **(B)** Distance of locomotor activity in the saline-paired compartment (black box) and in the propofol-paired compartment (white box) for the five doses of propofol. **P < 0.01 when compared to the saline group. **(C)** Place preference for the five doses of propofol. **P < 0.01 when compared to the 0 mg/kg group. All data are graphed as mean ± SEM.

The three consecutive phases of CPP were showed in Figure 2A. During the first 5 min of the conditioning session for the five doses of propofol, locomotor activity in the saline-paired compartment and in the propofol-paired compartment was recorded. No difference appeared in the locomotor activity in the saline-paired compartment between the five groups. Conversely, the locomotor activity in the propofol-paired compartment was significantly increased at the 4 mg/kg dose (57.58 ± 2.58 cm vs. 99.25 ± 4 cm, *P* < 0.01), but decreased at the 8 mg/kg doses (58.41 ± 3.2 cm vs. 4.16 ± 2.47 cm, *P* < 0.01) compared with that of the saline-paired group (Figure 2B). For the 8 mg/kg dose, a transient hypnosis occurred in nine of the 12 rats after propofol bolus injection. In Figure 2C, the results of post-conditioning test were presented. When compared with the 0 mg/kg group, the place preference increased in the 4 and 6 mg/kg groups, while the 4 mg/kg group showed the largest magnitude (56.42 ± 2.62 s vs. 25 ± 1.78 s, *P* < 0.01).

### 4 mg/kg of Propofol Increased the Neuronal Activity of Locus Coeruleus, Not the Ventral Tegmental Area

**FIGURE 3:**
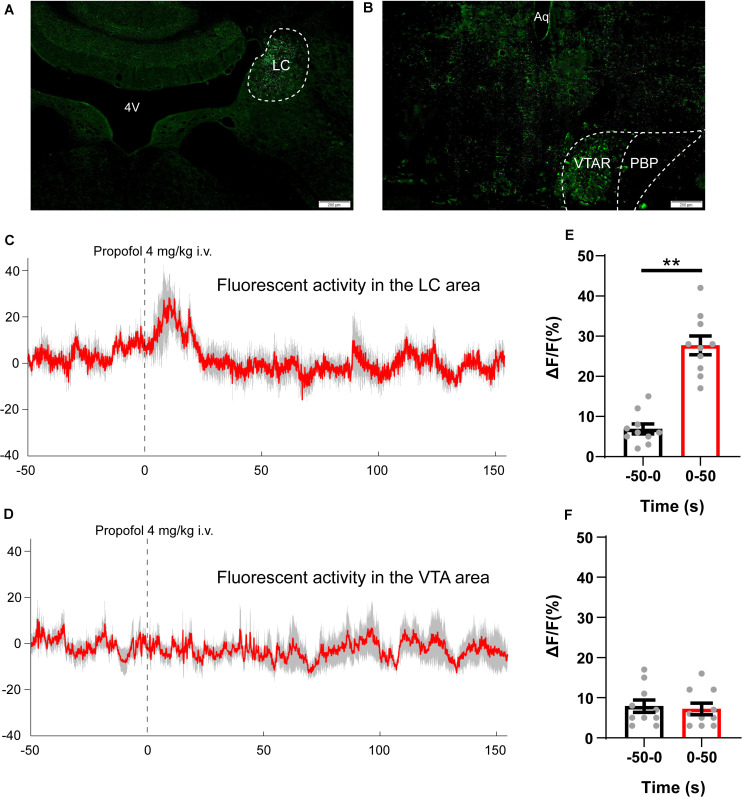
Propofol (4 mg/kg, i.v.) increases spontaneous activity of neurons in the LC area. **(A,B)** GcaMP expression in the **(A)** LC and **(B)** VTA area. Scale bar, 200 μm. 4V, 4th ventricle; LC, locus coeruleus; Aq, aqueduct; VTAR, ventral tegmental area rostral part; PBP, parabrachial pigmented nucleus of the VTA. **(C**,**D)** Averaged fiber photometry traces (red) from panel **(C)** LC GcaMP expression rats and **(D)** VTA GcaMP expression rats, gray area indicating the standard error of mean (SEM). **(E**,**F)** Column chart comparing the intensity of GcaMP signals (ΔF/F values) during 50 s pre-propofol and 50 s post-propofol periods. **P < 0.01 when compared to the −50–0 s group. All data are graphed as mean ± SEM.

GCaMP was expressed on the LC and VTA neurons as a calcium indicator to examine real-time neuronal activity. The AAV-hSyn-GcaMP6s virus was injected into the LC area or the VTA area (Figures 3A,B). Based on the results of hedonic facial response test and CPP experiments, 4 mg/kg of propofol induced the most obvious euphoric effects and, therefore, was chosen for the fiber photometry experiment. We analyzed the GcaMP signals in two periods: 50-s pre-propofol and 100-s post propofol tail-vein bolus injection. In the LC GcaMP-expressing rats, we observed that administration of 4 mg/kg of propofol triggers reliable increases in GcaMP fluorescence (Figures 3C,D, pre 6.9 ± 1.3 vs. post 27.7 ± 2.4, *P* < 0.01). However, in the VTA GcaMP-expressing rats, administration of 4 mg/kg of propofol did not induce any change in GcaMP signals (Figures 3E,F, pre 7.9 ± 1.6 vs. post 7.2 ± 1.4). Our results suggested that the euphoric effects of sub-anesthetic dose of propofol are mediated by the activation of LC neurons, not VTA neurons.

### Chemogenetic Inhibition of the Neuronal Activity in the Locus Coeruleus Area Eliminates the Hedonic Effects of Sub-Anesthetic Dose of Propofol

**FIGURE 4:**
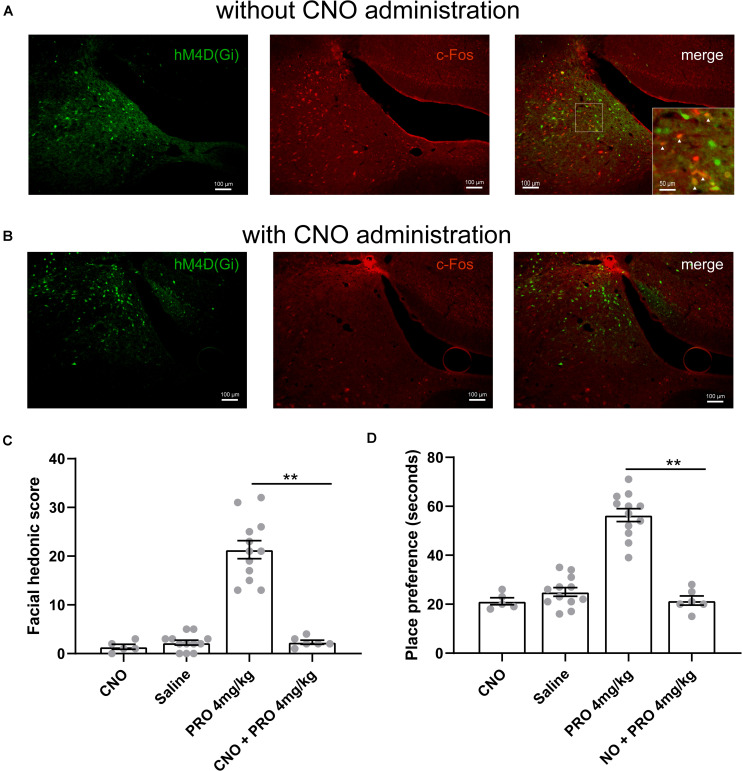
The hedonic effects of propofol (4 mg/kg, i.v.) are mediated by LC neurons. **(A)** hM4D(Gi) and c-Fos expression in the LC area of rat without clozapine *N*-oxide (CNO) (1 mg/kg) intraperitoneally injection. Scale bar, 100 μm. Inset showing higher magnification colocalized expression of hM4D(Gi) and c-Fos (yellow). **(B)** The c-Fos expression in the LC area was inhibited by CNO administration. **(C)** Hedonic facial responses were scored according to the facial reactivity scale. In the CNO group, five rats with control rAAV-hSyn-EGFP transfection received intraperitoneally injection of CNO. **(D)** Place preference for 4 mg/kg propofol with or without CNO pretreatment. In the CNO group, five rats with control rAAV-hSyn-EGFP transfection received intraperitoneally injection of CNO. **P < 0.01 when compared to the 4 mg/kg group. All data are graphed as mean ± SEM.

We next examined the influence of selective inhibition of neuronal activity in the LC area on the hedonic effects of sub-anesthetic dose of propofol. We delivered rAAV-hSyn-hM4D(Gi)-EGFP in bilateral LC of 30 rats. Four weeks later, hM4D(Gi) marked by GFP was verified in the LC area (Figure 4A). CNO (1 mg/kg) was intraperitoneally injected to selectively inhibit hM4D(Gi) expressing neurons in the LC area. The chemogenetic inhibition of neuronal activity was verified by a c-Fos staining (c-Fos and related immediate early gene products as markers of neuronal activity) in the LC area (Figure 4B). For hedonic facial response test, six rats received CNO injection 30 min before 4 mg/kg of propofol tail-vein bolus injection, then a 5-minute period of facial reactions was recorded and scored after propofol administration. The score of hedonic facial responses was not significantly different between the 0 mg/kg propofol group (2.25 ± 0.49, data in results section “Sub-anesthetic Dose of Propofol Induce Facial Hedonic Responses”) and CNO group (2.33 ± 0.42, Figure 4C). When compared with the 4 mg/kg propofol group (21.33 ± 1.84, data in results section “Sub-anesthetic Dose of Propofol Induce Facial Hedonic Responses”), the facial score was significantly inhibited in the CNO + 4 mg/kg propofol group (21.33 ± 1.84 vs. 2.33 ± 0.42, *P* < 0.01, Figure 4C). Hence, the hedonic facial responses induced by a sub-anesthetic dose of propofol can be inhibited by chemogenetic inhibition of the neuronal activity in the LC area. Another six rats were used for the CPP test. CNO was injected 30 min before 4 mg/kg of propofol (i.v.) and confined to the drug-paired compartment in the conditioning phase. As shown in Figure 4D, the place preference increased by 4 mg/kg of propofol (56.42 ± 2.62 s, data in results section “Sub-anesthetic Dose of Propofol Induce Facial Hedonic Responses”) was abolished by CNO administration (21.5 ± 1.84 s). CNO was intraperitoneally injected to the rats that received the control rAAV-hSyn-EGFP injection in the LC area to exclude the influence of viral vector and CNO on the results (Figures 4C,D). Such results suggest that inhibition of the neuronal activity in the LC area could inhibit the putative pleasant affective state induced by the sub-anesthetic dose of propofol.

## Discussion

The addictive potential of propofol has attracted the attention of clinical anesthesiologists for a long time (Cha et al., 2012; Wang et al., 2016a; Brechmann et al., 2018). However, previous studies looked into only one aspect of addiction such as reinforcing effect represented by conditioned place preference (Pain et al., 1996). In the present work, we used hedonic facial response scoring, conditioned place preference test, *in vivo* fiber photometry, and DREADDs to reveal that the hedonic effects of a sub-anesthetic dose of propofol are associated with increased neuronal activity in the LC, but not in the VTA.

Our results are consistent with a previous study that reported that a sub-anesthetic dose showed hedonic effects, which may be due to the change in balance between sedative and hedonic effects produced by different doses of propofol (Pain et al., 1996). However, in most animal studies on the euphoric or addictive effects of propofol, the drug was delivered by intraperitoneal injection (Pain et al., 1996; Tezcan et al., 2015; Wu et al., 2016), which is far different from the way the propofol is delivered in clinical practice. Therefore, in this study, we delivered propofol through tail-vein bolus injection to ensure that the onset speeds of propofol are similar to that in the clinic.

The facial response scale used in this study reflects the hedonic impact independent of motivation, which was invented by Berridge (2000) based on the facial response study on human infants, monkeys, and rats to reward stimulation. Locomotor activity is a commonly used physiological characteristic that reflects whether the central nervous system is excited or inhibited, sedative hypnotics such as diazepam can significantly reduce the locomotor activity of rats (Mendez-Cuesta et al., 2011), and stimulants of the central nervous system such as caffeine can increase the locomotor activity of rats (Alsufyani and Docherty, 2017). Conditional place preference test is a commonly used method to evaluate the dependence potential of drugs (Calpe-Lopez et al., 2019).

This is our first attempt to use facial reactivity scale to examine the hedonic effects of propofol. The variability of the facial responses of individual rats could prevent detection of subtle changes in hedonic response. Thus, we combined the facial response scale, locomotor activity, and CPP to assess the hedonic reactions of rats in response to four doses of propofol. We found that only rats that received 4 mg/kg of propofol increased the facial hedonic score and the locomotor activity, and showed significant place preference. These results indicate that propofol in a sub-anesthetic dose can induce solid hedonic effects and have dependence potential. The dependence potential of propofol has also been proved by previous studies (Pain et al., 1996; Cha et al., 2012; Lian et al., 2013). It is worth noting that all rats in the 4 mg/kg group did not show narcosis, and at the anesthetic dose (8 mg/kg), all hedonic effects of propofol shown in the facial hedonic score, locomotor activity, and CPP are gone, which suggests that propofol loses its hedonic effects or does not produce anhedonia at a dose enough to produce unconsciousness.

Using calcium fiber photometry recordings, we first found that the LC neurons displayed an attenuated GcaMP signal after tail-vein bolus injection of 4 mg/kg of propofol, but the GcaMP signal of VTA neurons was not altered, suggesting that the hedonic effects of a sub-anesthetic dose of propofol are associated with increased neuronal activity in the LC, but not in the VTA. Propofol is a GABAergic anesthetic, which potentiates GABA-induced Cl^–^ currents and, generally at higher concentrations, directly activate GABA_*A*_ receptors in the absence of GABA (Franks, 2008). The functional effects propofol has on GABA_*A*_ receptors can depend on the receptors’ subunit composition as well as on their distribution on the cell surface. The β3N265M mutation of the β subunit of GABA_*A*_ receptor was found to greatly change the narcotic effects of propofol (Jurd et al., 2003). In addition to the GABA_*A*_ receptors, the cyclic-nucleotide-gated (HCN) channels in the thalamocortical neurons are also found showing considerably higher sensitivity to propofol than it has in the hippocampus or in medullary neurons (Funahashi et al., 2004; Ying et al., 2006). Thus, a sub-anesthetic dose of propofol that increases the neuronal activity in the LC, not in the VTA, may be due to the intrinsic differences in subunit sensitivity and biophysical properties in the different brain regions.

Furthermore, the DREADDs–CNO-mediated inhibition of the LC neurons prevented the rewarding effects of propofol. Such results point to a major involvement of the LC in the hedonic effects of a sub-anesthetic dose of propofol, more specifically indicating that brain norepinephrine (NE) may have a critical role in the hedonic effects of a sub-anesthetic dose of propofol because the LC contains a relatively uniform type of neurons—norepinephrine-synthesizing neurons. The neuronal activity we recorded in the LC area mainly represents the activity of the noradrenergic neurons in the LC, and norepinephrine is a key neurotransmitter involved in emotion regulation and reward effects (Ventura et al., 2003). However, more specific genetic methods are needed to identify the downstream noradrenergic pathways that are associated with the rewarding effects of propofol.

In addition to being a target for propofol, the LC is believed to be a target for other addictive drugs such as opioids and cocaine (Van Bockstaele et al., 2010; Liu et al., 2014). The LC works as a relay station for peripheral autonomic input and exerts widespread innervation throughout the brain (Svensson, 1987). Many areas in the central reward system, including the VTA and amygdala, receive the LC-noradrenergic inputs (Flavin and Winder, 2013). Furthermore, the activity of the dopaminergic neurons in the VTA is reported to be mediated by noradrenergic neurons in the LC (Grenhoff et al., 1993), and lesions of the noradrenergic neurons in the LC are also shown to attenuate dopamine release in Acb (Lategan et al., 1990). However, the mechanisms of the activation of the LC neurons induced by a sub-anesthetic dose of propofol still need further study. Work with propofol showed that the neuronal activity of cortices was first reduced during slow propofol injection and that the loss of consciousness only occurred when the activity in the thalamus was abolished by propofol at a high dose (Bonhomme et al., 2001; Franks, 2008). Therefore, different brain areas show different sensitivity to propofol, and propofol at a sub-anesthetic dose may only inhibit the upstream nucleus that projects presynaptic inhibitory inputs to the LC, which leads to the activation of the LC neurons. Also, propofol at a sub-anesthetic dose may not be sufficient to induce GABA_*A*_ receptor-mediated hyperpolarization of these neurons.

In conclusion, the current study demonstrates that activation of the LC neurons contributed to the hedonic effects of a sub-anesthetic dose of propofol. Our data may have implications for the pharmacological treatment of propofol addiction in physicians and nurses (Monroe et al., 2011). Future studies are needed to investigate the specific noradrenergic pathways involved in the hedonic effects of propofol.

## Data Availability Statement

The original contributions presented in the study are included in the article/supplementary material, further inquiries can be directed to the corresponding authors.

## Ethics Statement

The animal study was reviewed and approved by Animal Care and Use Committees of Zunyi Medical University.

## Author Contributions

HC, DX, and JL carried out the experiment. HC wrote the manuscript with the support from YZ. WS helped to supervise the project. TY and JL conceived the original idea and supervised the project. All authors contributed to the article and approved the submitted version.

## Conflict of Interest

The authors declare that the research was conducted in the absence of any commercial or financial relationships that could be construed as a potential conflict of interest.
